# Individual Tree Structural Parameter Extraction and Volume Table Creation Based on Near-Field LiDAR Data: A Case Study in a Subtropical Planted Forest

**DOI:** 10.3390/s21238162

**Published:** 2021-12-06

**Authors:** Sha Gao, Zhengnan Zhang, Lin Cao

**Affiliations:** 1 Co-Innovation Center for Sustainable Forestry in Southern China, Nanjing Forestry University, Nanjing 210037, China; gaosha@nies.org (S.G.); zhangzhengnan@njfu.edu.cn (Z.Z.); 2 Nanjing Institute of Environmental Sciences, Ministry of Ecology and Environment of the People’s Republic of China, Nanjing 210042, China

**Keywords:** ULS, BLS, point cloud, merge, synergetic effects, forest structural parameters, taper equation, volume

## Abstract

Individual tree structural parameters are vital for precision silviculture in planted forests. This study used near-field LiDAR (light detection and ranging) data (i.e., unmanned aerial vehicle laser scanning (ULS) and ground backpack laser scanning (BLS)) to extract individual tree structural parameters and fit volume models in subtropical planted forests in southeastern China. To do this, firstly, the tree height was acquired from ULS data and the diameter at breast height (DBH) was acquired from BLS data by using individual tree segmentation algorithms. Secondly, point clouds of the complete forest canopy were obtained through the combination of ULS and BLS data. Finally, five tree taper models were fitted using the LiDAR-extracted structural parameters of each tree, and then the optimal taper model was selected. Moreover, standard volume models were used to calculate the stand volume; then, standing timber volume tables were created for dawn redwood and poplar. The extraction of individual tree structural parameters exhibited good performance. The volume model had a good performance in calculating the standing volume for dawn redwood and poplar. Our results demonstrate that near-field LiDAR has a strong capability of extracting tree structural parameters and creating volume tables for subtropical planted forests.

## 1. Introduction

A volume table is critical for forest resource inventory, forest growth monitoring, forest volume statistics, etc. [1]. Volume is a key metric for evaluating the effect of the cultivation of plantations, and acquiring the standing volume is a crucial foundation for precise plantation cultivation [2]. The accurate assessment of volume and the growth of planted forests are of great importance in maintaining regional and global forest ecosystems, as well as in scientific decision making concerning planation cultivation [3,4]. As the forestry sector standard in China, the local volume table (i.e., one-way volume table), which allows the estimation of the stem volume from the diameter at breast height (DBH), originated in the 1970s. However, due to changes in climate, site and forest resource structures in China, some volume tables from that year inevitably contain errors in the actual use process, which has a significant impact on the results of evaluating the volumes of plantations. Therefore, it is an urgent problem to redevelop the standard volume model with a high precision and a strong applicability and to compile a standard volume table (i.e., two-way volume table, requiring both DBH and tree height) for the precision of cultivation of planted forests depending on the different requirements for forestry production and forest cultivation in different areas. The forest volume is an important parameter for assessing forest health, as well as a crucial foundation for national and forestry workers when assessing the state of a forest and formulating cultivation strategies. Although volume tables have brought new opportunities to provide feasible and detailed volume estimations, using conventional field measurements to construct forest volume tables is still a challenging task that is labor-intensive, time-consuming, costly, destructive, and spatially limited [5,6,7]. Thus, a non-destructive approach is urgently needed to improve the efficiency and accuracy of estimations of forest volume for forestry workers in silvicultural practices.

In remote sensing, the emergence of light detection and ranging (LiDAR) technologies has provided non-destructive and three-dimensional (3D) measurements in order to consistently quantify forest structures across large areas at an unprecedented level of detail [8,9]. LiDAR technology can be operated from spaceborne, airborne, or near-field platforms, with each platform serving specific forest inventory needs [10]. Compared to spaceborne and airborne LiDAR systems, near-field LiDAR systems represent a promising alternative for characterizing more spatially detailed forest structures at the plot level and at regional scales due to their much lower cost, higher point density, and higher repeatability [11,12].

As a kind of near-field LiDAR remote sensing platform, unmanned aerial vehicle-borne laser scanning (ULS), also known as UAV-LiDAR, can integrate a low-cost LiDAR sensor with a UAV and offers an unsurpassed capacity to retrieve more accurate 3D structural representations of the forest canopy, including crowns, stems, branches, and understory vegetation [13,14,15]. In recent years, ULS has become particularly attractive in key development stages (e.g., planting, pruning, thinning, and harvest) of planation cultivation due to its light weight, low cost, wide availability, cloud insensitivity, and repeatability. Liu et al. [16] employed ULS metrics to predict forest structural parameters with parametric and non-parametric models and assessed the effects of UAV-LiDAR point cloud density on the derived metrics and individual tree segmentation results in a Ginkgo planation in southeastern China. Their results showed that k-NN performed well for predicting the volume (CV-R^2^ = 0.94, rRMSE = 8.95%). Li et al. [17] assessed and compared the performance of individual tree crown diameter estimation methods using a low-cost ULS and an airborne LiDAR separately in the same study area as in our study. The results showed that low-cost ULS obtained good results for individual tree crown diameter estimation (R^2^ = 0.806, RMSE = 0.195 m). Puliti et al. [15] employed a ULS system to estimate the tree volume of boreal forests with existing allometric models in southeastern Norway. They reported that the accuracy of the ULS estimates varied according to the forest structure, and it was highest in open pine stands and lowest in dense birch or spruce stands. Although many previous studies have also demonstrated the great potential of ULS for estimating forest structural parameters and its advantages compared to airborne LiDAR, these “top-down” ULS systems may be problematic in estimating the individual tree diameter at breast height (DBH) under the different sampling conditions due to attenuation of the laser beam when interacting with a very dense overstory and, consequently, the discontinuity and occlusion of the lower parts of canopy structures (e.g., stem diameter) [18,19,20,21,22].

Terrestrial laser scanning (TLS)—namely, ground-based LiDAR—also offers a detailed 3D representation of the surroundings of forest structures with millimeter accuracy [10,23,24]. Compared to conventional forest inventory tools, such as the use of hypsometers to measure tree height and tapes to measure tree stem diameters [25], the use of TLS point clouds provides non-destructive estimates of stem curve profiles and characterizes the branching structures of trees, thus further improving the modeling of the individual tree volume. TLS data can be collected with either a single- or a multiple-scan mode [24,26]. In a single-scan mode, the scanner is placed at a single point and the obtained data will, at best, make one side of an individual tree visible in the scan. However, this typical scan mode often results in occlusion problems due to the fact that lower branches, dense undergrowth vegetation, some stems, twigs, and leaves or other parts of the tree may not be scanned, as they are hidden by elements that are closer to the scanner [10,27]. To relieve these occlusion problems, a plot can be scanned with multiple scans at different positions instead of a single scan in a fixed position. Yrttimaa et al. [25] employed a multiple-scan TLS with point cloud cluster and RANSAC-cylinder filtering algorithms to quantify structural changes in boreal forests in Evo in southern Finland. Their results indicated that multiple-scan TLS has the capacity to characterize trees and forest stands in space and showed that TLS technology could estimate structural changes over time in boreal forests. Saarinen et al. [28] investigated the effects of TLS data captured at various distance (i.e., corresponding to 25%, 50%, 75%, and 100% of tree height) on the accuracy of the stem volumes derived. The results indicated that a scanning distance of approximately 25% of the tree height would be optimal for stem volume estimation. Theoretically, a more complete set of forest observations should lead to improved estimates for forest structures [25]. Thus, some studies have attempted to employ ULS and TLS data in order to obtain complete vertical forest structures [25,29]. When combined, TLS and ULS can compensate each other for their respective defects in characterizing the complete and continuous vertical structures of individual trees and forest stands. However, the positive effect of this combination is still limited because the inflexibility and the short functional range in TLS data acquisition obviously increase the labor and time costs [26,30,31].

As a novel type of portable LiDAR platform, backpack laser scanning (BLS) (i.e., backpack LiDAR) integrated with a simultaneous localization and mapping (SLAM) algorithm can gather multi-scanned point cloud data under forest canopies efficiently and flexibly, and those scanned point clouds can be instantly auto-matched together [32]. Another advantage of BLS is that it can acquire data efficiently and flexibly under various terrain conditions, by using a professional and high-quality scanning senor and a navigation system [30]. Thus, the BLS technology has a high capacity in terms of accessibility and route choice. Notably, the implementation of a BLS system within a forest benefits greatly from the usage of the SLAM algorithm, which enables accurate positioning of the scanner in the forest environment, which hampers global navigation satellite system (GNSS) signals. The SLAM algorithm combines the posture of the LiDAR and the constraint relationships between changes in posture to solve for the position of the point cloud [32]. Tang et al. [33] investigated an SLAM-aided positioning solution with point clouds collected by a small-footprint mobile LiDAR and demonstrated the potential of positioning and mapping with SLAM in forest inventories. To date, the SLAM algorithm has been incorporated into some commercial scanners (e.g., Zebedee, GeoSLAM Horizon, Gexcel HERON, and Kaarta Stencil systems), and this algorithm has been widely explored in some forests [34,35,36]. In summary, the current shortcomings of TLS-based approaches include the aforementioned tree occlusion and need for multiple scans, as well as the lack of available software for processing (for specific fields) and the limited capacity to provide the information of the upper canopy layer (especially the tree-top height) [34]. Compared with TLS, BLS is usually much lighter, more flexible, more portable, and more timely [37]. Hyyppä et al. [34] used a pulse-based backpack mobile laser scanner to estimate the stem curves of individual trees in the boreal forests of Finland. They reported that the total root-mean-square errors (RMSEs) of the extracted stem curves were 1.2 (5.1%) and 1.7 cm (6.7%) for the easy and medium plots, respectively. Oveland et al. [31] compared TLS, BLS, and HLS (handheld laser scanning) in terms of tree stem detection and tree stem diameter estimation. Their results showed that BLS obtained better performance for both tree detection and stem diameter estimation compared to TLS and HLS. In theory, point cloud data can be obtained by combining the ULS and BLS technologies, and the vertical structural parameters can be extracted more completely and accurately. As a result, it seems reasonable to assume that the combination of ULS and BLS data has immense potential in the construction of complete structural information for the trunk, branch, and crown, as well as in the realization of the stem curve and construction of the volume equation.

To the best of our knowledge, most previous studies focused on the use of one or two sensors (ALS/TLS combination or ULS/TLS combination) for forest structure characterization in temperate or boreal forests. The published studies that have focused on the synergetic usage of ULS and BLS data to extract individual tree structural parameters and to fit volume models to derive a volume table of a subtropical planted forest are few. Therefore, this study is the first to proposes a new framework for estimating stem curves and generating volume tables based on a non-destructive approach in a subtropical planted forest using ULS and BLS data. The specific objectives of our study are: (1) to implement individual tree segmentation algorithms on ULS and BLS data; (2) to extract tree-level forest structural parameters by combining matched ULS and BLS data; (3) to fit taper models, construct volume models, and compile volume tables for dawn redwood and poplar trees in a subtropical planted forest.

## 2. Materials and Methods

### 2.1. Study Area

The study area was located in the state-operated Dongtai Forest (the Yellow Sea National Forest Park) (32°33′–32°57′ N, 120°07′–120°53′ E), Jiangsu Province, close to the Yellow Sea, with an annual average temperature of 14.6 °C and an annual average relative humidity of 88.3%. This site belongs to the northern subtropical monsoon climate zone and has four distinct seasons, full sunshine, abundant rainfall (annual rainfall of 1050 mm), and an annual frost-free period of 220 days [38]. Dongtai Forest is located in an alluvial plain with flat terrain, an average elevation of 4.5 m, and an average slope of 0.04%. The area of Dongtai Forest is approximately 2239 ha, the forest cover is 85%, and the main forest species are dawn redwood (*Metasequia glyptostroboides*), poplar (*Populus deltoids*), and ginkgo (*Ginkgo biloba*). This forest in the study area consists of many regular stands. In a stand, only one tree species is present, and the ages of the trees in the stand are the same. On the inside of the stand, regular planting is performed with a fixed-line spacing. The poplar stands have line spacings in ranges of 3 to 6, 4 to 5, 3 to 8, and 5 to 8 m, while dawn redwood stands have row spacings in ranges of 3 to 4 and 4 to 6 m. The forest farm’s personnel routinely nurses and manages the young forest, thinning the interior and adjusting the stand structure.

### 2.2. Field Data Collection

**Figure 1:**
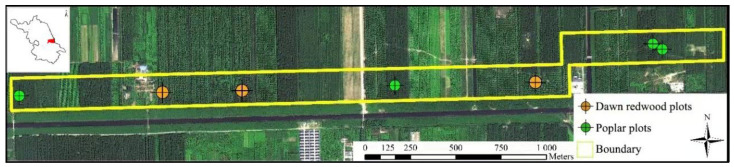
Study area and distribution of sample plots. The study area was located in Dongtai City in the east of Jiangsu province (see the subfigure in the top left) near the coast of the Yellow Sea.

In this study, a total of seven square plots with a 30 × 30 m^2^ area were designed and surveyed; these included three sample plots with dawn redwood and four plots with poplar. These plots’ stand densities were low, medium, and high. One additional sample plot of medium-density poplar was set up as a backup. The coordinates of the centers and corners of the plots were measured with differential GPS in each sample site. Figure 1 shows the distribution of the study area and sample plots. Field survey data were collected in Dongtai Forest from 23 to 26 July 2017. The survey factors within the seven plots included the trees’ species, single tree location information, DBH (>5 cm), and tree height. The position information of individual trees and the central coordinates and corners of the sample plots were obtained with real-time differential GPS (RTK, Real-Time Kinematic). The positions of the trees within the plots were measured using an ultrasound-based Haglöf PosTex^®^ positioning instrument (Långsele, Sweden). The DBH of the tree trunks was measured with a tape at a height of 1.3 m above the ground. The tree height was measured with a Vertex IV^®^ hypsometer (Långsele, Sweden). In this study, we only measured the DBH and heights of trees within a meter of each side of the walking route of the BLS data collection path (see Polewski et al. [39] for more details). Table 1 summarizes the structural attributes in the plots.

### 2.3. LiDAR Data Acquisition and Preprocessing

The full-coverage ULS data were acquired with a LiAir (GreenValley International, Berkeley, CA, USA) multi-rotor UAV-mounted VLP 16 (Velodyne 16E) laser sensor on 24–26 July 2017, with the unmanned aerial vehicle (UAV) integrated with the NovAtel SPAN-IGM-S1 Inertial Navigation System (inertial measurement unit, IMU) (NovAtel Inc., Calgary, AB, Canada). The UAV-LiDAR system flew at 86 m above ground level with a flight speed of 3.6 m/s. The laser wavelength emitted by the LiDAR was 903 nm, the scanning angle was 15°, the beam divergence was 3 mrad, the number of independent layers of Velodyne 16E was 16 line/s, and the pulse emission frequency was 21.7 kHz. The final LiDAR point cloud data acquired had an average point cloud density of about 84 pts/m^2^, and the average number of ULS point clouds in each sample plot was approximately 270,000 pts. The IMU had a gyro (bias instability of 0.5 deg/h, input range of 400 deg/sec, angular random walk of 0.15 deg/√hr) and accelerometer (bias instability of 0.05 mg and range of 10 g) with a random walk velocity of 0.06 m/s/√hr.

The full-coverage BLS data of the seven plots were obtained with the LiBackpack (GreenValley International, USA) mobile LiDAR backpack system on 23–26 July 2017. For the BLS data of each plot, four reference poles were placed at the beginning and end of two outer strip paths. An “S”-shaped strip path (see Polewski et al. [39] for more details) was designed to collect the BLS point clouds for the plots. The walking speed was 1 m/s. The vertical scanning angle of the instrument was ±15°, the laser wavelength was 905 nm, the divergence was 3 mrad, the pulse emission frequency was 21.7 Hz, the scanning frequency was 16 lines/s, the height of the scanner was 1.9 m, and the average number of BLS point clouds in each sample plot was approximately 5.7 million pts.

Because non-vegetation objects, such as birds in the sky, that obstructed the laser pulse would interfere with the point clouds in the raw LiDAR data, it was necessary to preprocess the data. Firstly, the ULS and BLS point cloud data were de-noised to remove noise points that were not associated with the forest. The improved progressive TIN densification (IPTD) filter algorithm was adapted from Zhao et al. [40] and used to filter and extract the aboveground points. After filtering the aboveground points, a 1-m digital elevation model (DEM) was generated by calculating the average elevation from the ground of points within a rasterized cell grid using the ground points of the ULS data with approximately 8000 pts per plot. If there were no returns within cell grids, these cell grids were filled through interpolation by using an inverse-distance-weighted (IDW) algorithm. Then, the point cloud height value was normalized against the DEM surface height. The normalized point cloud data were obtained by subtracting the elevation information of each point in the point cloud data from the elevation value vertically projected onto the DEM. After normalization, the elevation value of the point cloud data was the height value of each point’s actual distance from the ground.

### 2.4. Tree Height Extraction with ULS Data

The individual tree segmentation algorithm in this study was an algorithm from Li et al. [41]. The individual tree segmentation technique used in this work was based on the point cloud distance judgement clustering approach in order to extract the crown widths of single trees, and the algorithm had a good segmentation effect in a mixed coniferous forest in the Nevada mountain range in the United States [41]. The algorithm was directly based on a point cloud, and the steps of the process were as follows: The highest point of the normalized point cloud data was searched within a certain threshold value T_1_ in the point cloud data of the experimental area; each high point was treated as a treetop point, and the threshold value was close to the spacing between two trees. The other threshold value T_2_ was set, and the points outside the threshold of each treetop point as the center of the circle were not divided into the sets of points of the tree; the threshold value was close to the average crown radius of the sample. From the top to the bottom of the tree, the points in the threshold T_1_ were determined and segmented in turn. According to the minimum distance rule, the points were divided into the sets of points generated by trees with relatively close horizontal distances. To ensure the accuracy of the segmentation, the shape index of the branches was added. According to this method, all of the points above a certain height were determined and segmented from top to bottom, and the points of the entire group were divided into several parts to achieve the goal of single-tree segmentation in the study area.

The position information of the single field-measured trees in the seven samples was used to assess the accuracy of the single-treetop information detected in the ULS data. It was deemed correct when a detected tree was located within the crown of the field inventory tree. The recall (*r*, represents the detection rate), the precision (*p*, represents the precision of detected trees), and the overall accuracy (*F*, represents the overall accuracy, taking both omission and inclusion into consideration) were the three indexes that reflected the effects of individual tree segmentation:

(1) $$r=\frac{N_{t}}{N_{t}+N_{o}},$$

(2) $$p=\frac{N_{t}}{N_{t}+N_{c}},$$

(3) $$F=\frac{2\left(r\times p\right)}{r+p},$$

Among them, *N*_t_ is the number of detected treetops that existed in the field position, *N*_o_ is the number of the trees that were omitted by the algorithm, and *N*_c_ is the number of the detected trees that did not exist in the field.

After the point cloud data were segmented, they could be divided into different single trees; the point cloud of the same single tree could have the same ID information, and the highest point in each individual tree’s point cloud would be regarded as the top of the tree. The average horizontal distance between all point clouds was divided for each single tree, and the apex of the tree was regarded as its crown radius. Taking the top of the tree as the circle’s center, the relative value of the vertical height from the ground of the point where the center of the circle was located was taken as the tree height information of the single tree. In each sample, all accurately segmented single tree heights were retrieved, and the corresponding IDs were created.

### 2.5. DBH Extraction with BLS Data

The algorithm for single-tree segmentation of the trunk was a clustering algorithm that was directly based on the density of the point cloud. After the LiDAR point cloud data were subjected to de-noising and preprocessing with normalization, the point cloud data were horizontally sliced, and then a threshold value of the density of a point set was determined to detect the presence of the trunk. In this study, a clustering algorithm—namely, density-based spatial clustering of applications with noise (DBSCAN)—was adopted for trunk segmentation because of its robustness to noise points and efficiency [37]. The method comprised the following steps: slicing the normalized LiDAR data at a height of 1.3 m and a vertical width of 10 cm and using the DBSCAN algorithm to detect the tree trunk. This algorithm could automatically detect clusters of points of different shapes after setting the minimum number of points (MinPts) for the trunk point set and the threshold radius (*Eps*) of the points. The point set *D* was a set of points in the *p*-point threshold range; the *q*-point belonged to the point set *D*, and the number of points of the set of points *q* (including the *p*-point itself) was represented by *N_Eps_* (*p*), i.e.,

(4) $$N_{Eps}\left(p\right)=\left\{q\in D/dist\left(p,q\right)\leq Eps\right\}.$$

If there was a sufficient number of points in this neighborhood, i.e., |*N_Eps_*(*p*)| ≥ MinPts, it was considered that a trunk was detected. Otherwise, the point *q* was considered as a noise point. This step was repeated for each point in the point cloud after the slicing. Finally, an analysis of the accuracy of the individual tree segmentation with the BLS was performed by utilizing the position information of the single trees that were measured in the seven samples. The segmentation accuracy was expressed by using the *r*, *p*, and *F* indices in the upper section.

After performing tree trunk segmentation on the cloud data, a set was formed with the point cloud of the same tree and was sliced at a distance of 1.3 m from the ground, the distribution of the point cloud was fitted by using a circle, and the diameter of the circle was considered as the detected breast diameter. The steps in the procedure were as follows: calculating a gravity center of all points of a point set at a point at 1.3 m after slicing, taking the average distance of all points in the point set from the center of gravity as a radius, and calculating the DBH of the tree.

### 2.6. Co-Registration of ULS and BLS Data

In this study, control of coordinate points was used to coarsely calibrate the ULS data and BLS data. The method of controlling coordinate points was adopted for the rough calibration of the two types of data. The UAV-LiDAR system had a global navigation satellite system (GNSS) and an inertial navigation system (IMU), which recorded accurate coordinate information in real time, so the point cloud in the ULS data had absolute coordinates; while the working environment of the mobile backpack laser scanner was below the forest canopy, even if equipped with GPS, the real-time coordinate information obtained was also unreliable due to the canopy occlusion and weak GPS signal, so the BLS data had only relative coordinates. The goal of coarse calibration was to roughly match the two types of source data into one coordinate system in preparation for fine calibration. The method was as follows: TrimbleNet R9 (Trimble, Sunnyvale, CA, USA) was used as the base station, and the coordinate points of the base station used the coordinate points of the ULS coordinate system to ensure that the coordinates of the mobile station were consistent with the coordinate system in the ULS data; Trimble R4 (Trimble, Sunnyvale, CA, USA) was used as a real-time kinematic (RTK) positioning system rover to receive the GNSS and the differential signal of the base station. The coordinates of four reference poles (height of 2 m) were measured using this RTK positioning system (error ≤ 3 cm). The control points were the spatial coordinates of the top positions of the four poles on the sample edge, and the approach of controlling coordinate points was used to coarsely register the BLS and ULS point cloud data.

Polewski et al. [39] presented an object-oriented point-cloud-matching algorithm based on relative position information between trees, which was applied in this study for the fine co-registration of the ULS and BLS data. The algorithm was utilized for fine co-registration using the positional relationship between the ULS-detected treetop point and the BLS-detected tree trunk, and it required the position information of the treetop point and the tree trunk. Geographic coordinates are required for point cloud data acquired by BLS in common forest research [42]. Therefore, the completion of fine co-registration of ULS and BLS data has become an essential process. Currently, the majority of point-cloud-matching algorithms rely on the detection of identical points at the 3D level for the transformation of the coordinate system [43,44]. Because finding the same feature point clouds for ULS and BLS is difficult, this study used a complete automation algorithm based on the location information of the treetops and tree trunks extracted from the point cloud data; an algorithm for point cloud matching between trees was used to complete the fine co-registration of the ULS and BLS in the forests.

### 2.7. Extraction of the Diameter of the Upper Part of the Trunk

The height of the BLS data obtained in the 30 × 30 m^2^ sample plots was limited due to the scanning angle constraint of the BLS sensor and could only reach a height of roughly 9 m. In this study, BLS-derived trunks ranging from 1.3 to 8.3 m were sliced in height intervals of 1 m. The diameters of the trunk at 1.3, 2.3, and 3.3 m up to 8.3 m were fitted, and the diameter information at different heights above the DBH of each tree was obtained. The different heights of each single tree were compiled with the same ID.

### 2.8. Construction of the Taper Equation

The tree species in this study were dawn redwood and poplar, and the trunks of both are straight, so it was not necessary to segment the trunks. The following five taper equations were chosen as alternative models by referring to previous studies—those of Ormeod [45], Demaerschalk [46], Meng [47], Kozak [48], and Yan Ruohai [49]—and the taper equations were all converted into the same form:

(5) $$d^{2}=f\left(D,H,h\right),$$

The following five alternative taper equations were used for fitting using the LiDAR-derived structural parameters, such as tree height, DBH, and upper trunk diameter, and finally, the group with the highest accuracy was selected as the taper equation model.

(6) $$d^{2}=D^{a_{0}+a_{1}H}{\left(H-h\right)}^{a_{2}+a_{3}D}/H,$$

(7) $$d^{2}=a_{0}D^{a_{1}}\frac{{\left(H-h\right)}^{a_{2}}}{H^{a_{3}}},$$

(8) $$d^{2}=a_{0}D{\left(\frac{H-h}{H-1.3}\right)}^{a_{1}},$$

(9) $$d^{2}=D^{2}{\left(\frac{H-h}{H-1.3}\right)}^{a_{0}},$$

(10) $$d^{2}=D^{2}\left(a_{0}+a_{1}\frac{H-h}{h}\right),$$

Here, *D* is the DBH, *H* is the tree height, *h* is the tree trunk height, *d* is the diameter of the tree trunk at height h off the ground, and *a*_0_, *a*_1_, *a*_2_, and *a*_3_ represent parameters. Through the co-registration of the ULS and BLS data, each tree in the seven plots had a mapping relationship between the DBH and diameter of the upper part of the trunk derived from the BLS data and the height derived from the ULS data. These structural parameters were input into five alternative taper equation models (Equations (6)–(10)); a multivariate nonlinear regression analysis was carried out by using the Levenberg–Marquardt iteration method, and the parameters of five taper models were obtained. The RMSE was used to compare and analyze the diameters at different heights extracted from the LiDAR data and the diameters at the same height in the taper equations, and it was used as an index to evaluate the advantages and disadvantages of the taper equations. The diameter of the upper part of the trunk extracted from the LiDAR point cloud data was used as the real value, and the diameter of the upper part of the trunk fitted by the taper equations was used as the observation value.

### 2.9. Compilation of a Standard Volume Table

The taper equation denotes the relationship between the diameter of a trunk and its height. The taper equation can describe the trend of change in the trunk’s shape, and it is the basic method for studying the outputs of trees. After the evaluation of the taper model, the optimal taper equation was integrated, and the corresponding standard volume equation could be obtained.

(11) $$V=K\int_{0}^{H}d^{2}dh=K\int_{0}^{H}f{\left(h,H,D\right)}^{2}dh,$$

where *K* = π/40,000, which is a function of diameter *d* at different heights of the trunk. Based on the stand volume model obtained for the two tree species, the stand volume of the corresponding tree height and DBH was calculated, and a standard volume table suitable for the dawn redwood and poplar species in Dongtai Forest was compiled.

### 2.10. Volume Calculation

The method for determining the standing volume in this study was as follows: The mean diameter (D_g_) of the sample plot was calculated by using the DBH information obtained from the BLS data, and the average tree height (H_g_) in the sample area was calculated by using the tree height information obtained with the ULS data. According to the D_g_ (1 ± 5%) and H_g_ (1 ± 5%) in the sample area, we selected the multi-plant standard tree and used the integral to obtain the volume of the material; then, we calculated the volume of the sample plot and the cumulative stand volume per unit area (m^3^/ha).

(12) $$M=\sum_{i=1}^{n}V_{i}\frac{G}{\sum_{i=1}^{n}g_{i}},$$

where *M* is the volume of the sample plot, *n* is the number of standard trees, *g_i_* is the basal area of standard tree *i*, *V_i_* is the volume of standard tree *i*, and *G* is the total cross-area of the sample plot.

## 3. Results

### 3.1. Single-Tree Segmentation Results

#### 3.1.1. Single-Tree Segmentation with ULS Data and Accuracy Assessment

**Figure 2:**
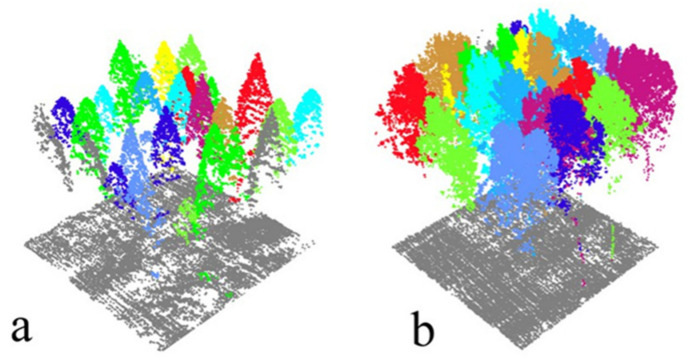
Single-tree segmentation results with the ULS data; point clouds of the same color show the same tree. (**a**) The segmentation results of a dawn redwood plot; (**b**) the segmentation results of a poplar plot.

The individual tree segmentation with the ULS data was carried out with the distance-discriminant clustering method oriented to point clouds (Table 2). The overall accuracy (*F*) of the individual tree segmentation for dawn redwood was 0.90, and that for poplar was 0.88. The ULS segmentation results are shown in Figure 2; the point clouds of different colors are different single trees. It can be seen in Figure 2 that the crown information of the ULS data was relatively complete, but the point cloud data below the forest canopy were insufficient to show the vegetation information of the trunks and lower layer. The gray point clouds in Figure 2 show the ground points. Poplar is a broad-leaf tree species because of the large bifurcations of the crown branches, but the leaves are not as dense as those of dawn redwood. Therefore, there were more ground points for the poplar plots.

#### 3.1.2. Single-Tree Segmentation with BLS Data and Accuracy Assessment

The results of the assessment of the accuracy of individual tree detection with the BLS data are shown in Table 3. In comparison to poplar, dawn redwood resulted in a slightly higher segmentation performance, as demonstrated in Table 3. In terms of overall accuracy, three dawn redwood plots obtained a mean *F* value of 0.93, while the four poplar plots had a mean *F* value of 0.92. In addition, we can see that other trees that did not exist in the field might have been detected in the poplar plots, especially in Plots d (*p* = 0.89) and g (*p* = 0.89).

### 3.2. Accuracy Assessment of LiDAR-Derived Tree Height and DBH

**Figure 3:**
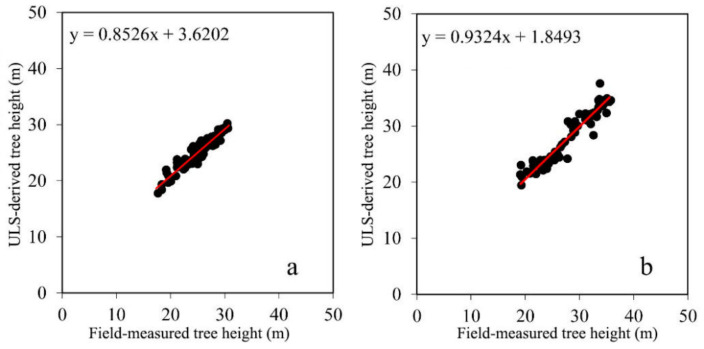
Scatterplots of ULS-derived and field-measured tree height. (**a**) Dawn redwood, (**b**) poplar.

**Figure 4:**
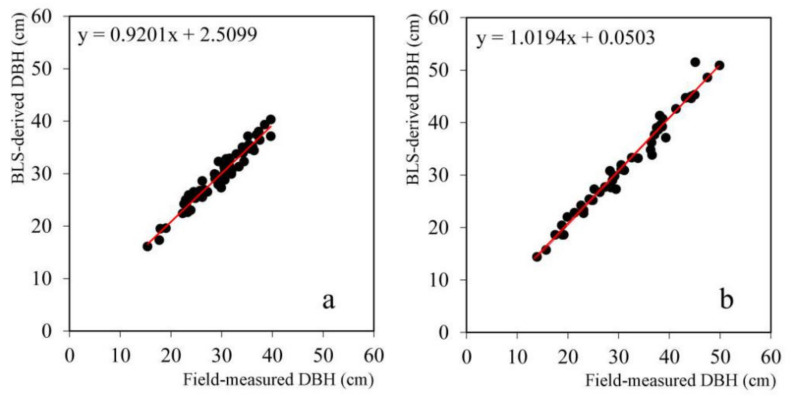
Scatterplots of the BLS-derived and field-measured DBH. (**a**) Dawn redwood, (**b**) poplar.

Scatterplots between the field-measured and ULS-derived results for the tree height are shown in Figure 3. Figure 4 shows scatterplots of the field-measured and ULS-derived DBH results. The accuracy of the tree height and DBH retrieved from the LiDAR data is shown in Table 4. According to Figure 3 and Table 4, the R^2^, RMSE, and rRMSE of the height predicted for dawn redwood were 0.913, 0.849 m, and 3.00%, respectively. For poplar, the R^2^, RMSE, and rRMSE of the predicted height were 0.934, 1.246 m, and 4.40%, respectively. The R^2^, RMSE, and rRMSE of the DBH for dawn redwood and poplar were 0.945 and 0.97, 0.013 and 0.016 cm, and 4.5% and 4.9%, respectively.

The error in the tree height detected with the ULS data was within an acceptable range compared with the actual tree height, in which the error in the height detection for dawn redwood was lower than that for poplar, and the accuracy of the ULS-derived tree height for the dawn redwood (rRMSE = 3.00%) was slightly better than that for poplar (rRMSE = 4.40%). The RMSE of the DBH extracted from the BLS data (dawn redwood: RMSE = 1.3 cm, poplar: RMSE = 1.6 cm) was below 2 cm, which could meet the requirements of forestry research. In addition, the accuracy of the BLS-derived DBH of the dawn redwood (rRMSE = 4.50%) was better than that of poplar (rRMSE = 4.90%). For the dawn redwood and poplar species, the detection accuracy of the tree height was higher than that of DBH.

### 3.3. Co-Registration of ULS and BLS Data

In this study, a co-registration of ULS and BLS data was performed in order to obtain the relatively complete vertical structures of individual trees. Table 5 presents the effects of matching with rough co-registration by measuring the horizontal distance between the apex of a tree and the trunk point. The error distance for dawn redwood was less than 1 m, and the coarse calibration error for high density was lower than that of for density; the rough calibration error for Poplar was generally higher, and it reached more than 1 m.

For the two tree species, the matching precision for the dawn redwood was obviously higher than that for the poplar, and the position error was lower than that for the poplar. The reason for this may be due to the fact that the tree trunks of the dawn redwood were basically vertical with respect to the ground, the horizontal coordinates of the trunk and the top of the tree were not large, and the tree crown of the poplar was extended—the highest point of the top of the tree could deviate from the tree trunk in the horizontal direction, so the matching precision for poplar was not good.

### 3.4. Construction and Selection of Taper Equations

**Figure 5:**
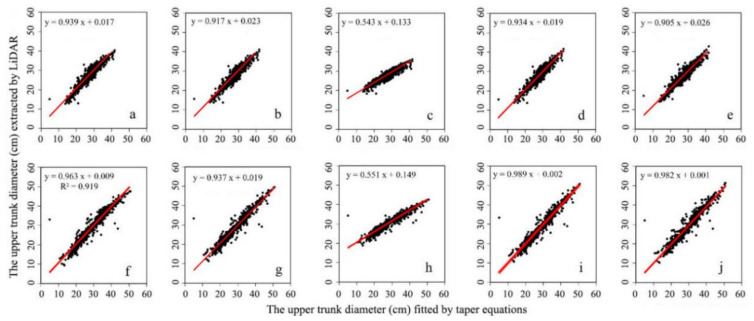
Scatterplots of the upper diameter of the trunk and the upper diameter of the trunk extracted using the LiDAR data for the five taper equation models for dawn redwood and poplar. (**a**–**e**) Dawn redwood, (**f**–**j**) poplar.

**Figure 6:**
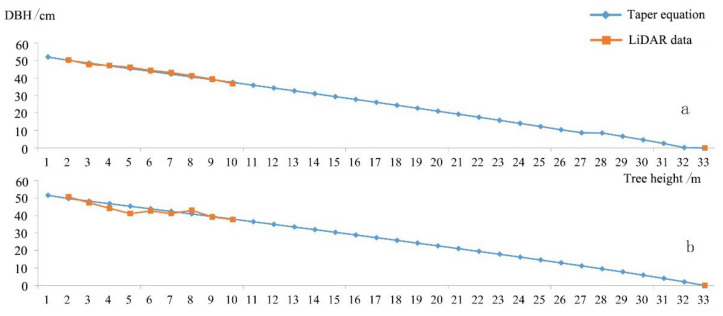
Comparison of the stem curves of dawn redwood and poplar acquired from taper model and LiDAR data. (**a**) Dawn redwood, (**b**) poplar.

The diameters at different heights extracted from the LiDAR data and the diameters at the same height that were fitted in the taper function models were compared and analyzed with 1097 samples (786 samples of dawn redwood and 611 samples of poplar). The results of the analysis are shown in Figure 5 and Table 6. As shown in Table 6, models ➀, ➁, ➃, and ➄ were suitable for the growth laws of dawn redwood and poplar, and model ➁, the improved Schumacher model, had the greatest goodness-of-fit and higher accuracy. From Figure 5c, we can see that the slope of the scatterplot of model ➂ was not obviously close to the 1:1 line, and the RMSE values for dawn redwood (RMSE = 2.8 cm) and poplar (RMSE = 4.2 cm) in model ➂ were obviously larger than those in the other four models; the rRMSE values were also approximately 10%. For poplar, taper model ➂ reached 13.6% in comparison with the other models. Taper model ➂ was not suitable for studying the growth laws of dawn redwood and poplar. The performance of model ➁ was the best, so this model was selected for the study of the volumes and growth laws of dawn redwood and poplar. Model ➁ was an improved Schumacher model, which is often used to study the growth laws of poplar stands [50]. In this study, single trees with the same tree height were selected for dawn redwood and poplar. The diameters extracted with the LiDAR data for these two tree species were compared with the results produced by model ➁, and the results of the comparison are shown in Figure 6. It can be seen in Figure 6 that model ➁ could roughly represent the trunk shapes of the two tree species, and the taper model of dawn redwood in Figure 6a was more consistent with the LiDAR-produced results than that of poplar.

The coefficient parameters of the taper equations for dawn redwood and poplar calculated according to the Levenberg–Marquardt iteration method were brought into the optimal taper equations, and the optimal taper equations of the two tree species were obtained (Table 7). The optimal taper equation of dawn redwood is as follows:

(13) $$d^{2}=0.598D^{1.9}\frac{{\left(H-h\right)}^{1.417}}{H^{1.279}},$$

The optimal taper equation of poplar is as follows:

(14) $$d^{2}=1.501D^{1.901}\frac{{\left(H-h\right)}^{1.724}}{H^{1.854}},$$

According to the results of the above analysis and evaluation, taper model ➁, $d^{2}=a_{0}D^{a_{1}}\frac{{\left(H-h\right)}^{a_{2}}}{H^{a_{3}}}$, was chosen as the most suitable taper model for two tree species.

### 3.5. Construction of Standard Volume Models and Validation and Compilation of Standard Volume Tables

The equation of the correlation of the two factors of the tree height and DBH with the tree volume is called a standard volume equation. By integrating the above-mentioned model of the taper equation, the standard volume equation of the tree volume *V* with respect to the DBH and the tree height can be obtained. The model is integrated and the following general form of the standard volume model is obtained:

(15) $$V=\frac{Ka_{0}D^{a_{1}}}{\left(a_{2}+1\right)H^{a_{3}}}H^{a_{2}+1},$$

where *K* = 1/40,000; *V* represents the tree volume, *D* represents the DBH derived from the BLS data, and *H* represents the tree height derived from the ULS data. According to the parameters of the optimal taper model of the dawn redwood and poplar, the standard volume model for the dawn redwood can be obtained as follows:

(16) $$V=\frac{K\cdot 0.598\cdot D^{1.9}}{\left(1.417+1\right)H^{1.279}}H^{1.417+1},$$

The standard volume model for poplar is as follows:

(17) $$V=\frac{K\cdot 1.501\cdot D^{1.901}}{\left(1.724+1\right)H^{1.854}}H^{1.724+1},$$

**Figure 7:**
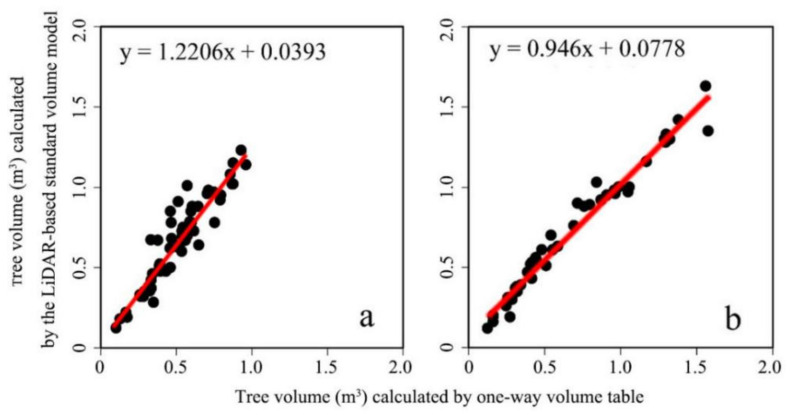
Tree volumes calculated with the LiDAR-derived standard volume equations of dawn redwood and poplar vs. tree volumes obtained by finding them in the local single-entry volume table. (**a**) Dawn redwood, (**b**) poplar.

To verify the accuracy of the tree volume equations obtained, the tree volumes of validation samples (59 dawn redwood trees and 47 poplar trees) were calculated with Jiangsu Province’s existing single-entry volume table, and the results were compared with the corresponding volumes from the standard volume models for the two species. The tree volume, which was found with the volume table as the real value and the volume from the LiDAR-generated standard volume equation, was used as the observation value, and the results were analyzed and compared by means of the R^2^, RMSE, and rRMSE. The results of the comparison of the tree volumes obtained from the local single-entry volume table and the LiDAR-generated standard volume models for dawn redwood and poplar are shown in Table 8. In the comparison results, the standard volume model for dawn redwood returned values of R^2^, RMSE, and rRMSE of 0.868, 0.058 m^3^, and 11.2%, respectively. The standard volume model for Poplar returned values of R^2^, RMSE, and rRMSE of 0.97, 0.022 m^3^, and 3.3%, respectively. The results showed that the standard volume equations could be used as the bases for the estimation of the standing volumes of dawn redwood and poplar to a certain extent. As shown in Figure 7, the tree volumes calculated with the standard volume equations for the two species in this study had a slight tendency toward the tree volumes found in the existing single-entry volume table. In terms of the RMSE and rRMSE, it can be seen that the RMSE of the poplar was smaller and the rRMSE was lower, so the standard volume equation was more suitable for the poplar than for the dawn redwood.

According to the standard volume equations obtained in this study, the local standard volume tables for dawn redwood and poplar could be calculated. Tables S1 and S2 (in the Supplementary Materials) are the standard volume tables of dawn redwood and poplar, respectively.

### 3.6. Stand Volume Calculation

As indicated in Table 9, the plot volumes of the dawn redwood from low stem density to high stem density were 19.15, 33.42, and 16.39 m^3^. The plot volumes of the poplar from low stem density to high stem density were 10.46, 21.75, 24.33, and 10.42 m^3^. The stand volumes of the three dawn redwood plots from low stem density to high stem density were 212.78, 371.33, and 182.11 m^3^/ha, respectively. The stand volumes of the four poplar plots were 116.22, 241.67, 270.33, and 115.78 m^3^/ha, respectively.

## 4. Discussion

In this study, the distance judgment clustering algorithm directly oriented toward point clouds was employed to segment crowns in ULS data, and the overall segmentation performance was better (*F* of dawn redwood = 0.90; *F* of poplar = 0.88). This segmentation was also implemented by Gao et al. [51], where the single-tree segmentation of five plantation species was carried out in Yushan Forest, Changshu, Jiangsu Province; their detection rate was 85.7%, the accuracy rate was 96%, and the overall accuracy was 90.9%. Point cloud data can directly reflect the structural information of a forest canopy, but traditional individual tree segmentation based on remote sensing images cannot obtain the full structural information of a forest, so the segmentation accuracy of individual tree segmentation using LiDAR point cloud data is usually higher than that of traditional remote sensing image extraction. Shen et al. [52] extracted the crowns and crown widths of single trees from a subtropical secondary forest with high-resolution remote sensing images, and their detection rate was 77.3%, the accuracy rate was 85.9%, and the overall accuracy was 81.4%. In this study, the density clustering algorithm directly oriented toward point clouds was used to segment trunks in BLS data, and the segmentation accuracy was high (*F* of dawn redwood = 0.93; *F* of poplar = 0.92). When a clustering algorithm based on point cloud density was used for individual tree segmentation, it was necessary to reasonably set the threshold of point density. If the threshold was too large, part of the trunk would not be recognized. If the threshold was too low, the phenomenon of tree trunk misclassification would occur, and many irrigated grasses with a height of 1.3 m would be mistakenly classified as trunks. As a result, it was necessary to adjust the threshold in the trunk segmentation process on a regular basis to achieve a better segmentation effect. In this study, the BLS collection route was “S”-shaped, and the planting rules (e.g., same space, etc.) were regular. The design of this route could completely cover the trunks of all of the trees standing in the sample plot, resulting in a more complete trunk point cloud and more accurate DBH data.

For the coarse co-registration of the ULS and BLS data, the method of controlling coordinate points was adopted. This method does not need to find the points with the same name in the two types of data to perform matching. In the experimental design, considering the data fusion of the two sources in the later stage, the coordinate information of the RTK base station was kept while a UAV positioning base station was set up, which could have caused the two groups of RTK data (one set of UAV data and one set of handheld data) have the same real-time coordinate system. Because it is usually difficult for GPS signals in a forest to penetrate the crown, it is difficult to locate single trees directly. In this study, the GPS signals were accepted through forest gaps for high-precision positioning, and then the coordinate information of each individual tree and marking rod was measured with the coordinate transformation method. The lengths of poles inserted into soil were also taken into account when calculating their coordinates, which also ensured the accuracy of the rough calibration. In the process of fine matching of the two types of source data, an object-oriented matching method based on the relative positions of the trees was used in this study. The matching accuracy and distance error could be improved by 20% and 13% in our study area, which was reported by Polewski et al. [39].

The traditional method of studying taper equations and standard volumes requires cutting down some standard trees, which is time-consuming and labor-intensive; it will also ruin the forest to a certain extent, and the taper model and the estimation of the growth conditions will be delayed. Therefore, this study proposed a non-destructive approach to constructing LiDAR-derived taper equations, which can effectively avoid the disadvantages of traditional methods. Using LiDAR point cloud data to obtain the tree volume models of planted forests can result in quickly obtaining tree volumes and stand volumes without causing damage to forests, and the error and accuracy of the volumes will also be within the acceptable range of error for forestry research work. The error of the predicted volume of dawn redwood was more than 0.05 m^3^, while that for poplar was only 0.02 m^3^. It was found that the volume equation was more suitable for the poplar. The growth of poplar in Dongtai Forest Farm is normal, which is in accordance with the growth law of poplar in Jiangsu Province. The LiDAR-derived standard volume model of dawn redwood was lower than the volume calculated with the Jiangsu single-entry volume table, and the Dongtai Forest Farm may be located on a coastal alluvial plain. Compared with the other areas of Jiangsu that are not coastal alluvial plains, the saline–alkali content in soils is higher, and the effect of inhibition on the growth of dawn redwood in the Dongtai Forest Farm is also affected.

Multisensorial approaches are capable of improving the full characterization of forest structures [25]. Bazezew et al. [53] attempted an integrative use of ALS and TLS to estimate AGB and carbon stock by integrating ALS- and TLS-derived forest parameters in the Ayer Hitam tropical rainforest of Malaysia. Their results showed that the integrative use of ALS and TLS was able to enhance the estimation of AGB (or carbon stock) in the tropical forests. The feasibility of combining ALS and TLS to obtain more complete descriptions of forest structures has been demonstrated in other studies [53,54,55,56]. However, such combinations of ALS and TLS still have some limitations [25,57,58]. The range of data acquired with ALS usually covers the entire study area. However, due to the complexity of forest conditions, the range of TLS data is often limited in terms of the sizes of specific plots, which necessitates a large amount of work when selecting plots in a study to ensure that the selected plots are representative of the entire forest stand. If the situation in a forest is extremely complicated and TLS is difficult to operate, it will be preferable to derive the information in the situation under and inside the treetops by using ALS with a high density and level of penetration in order to obtain the parameters of the relatively complete forest canopy structure. Based on existing knowledge, it is known that ULS and BLS data are usually obtained with higher flexibility and at a lower cost and can provide higher point density compared to traditional ALS and TLS, and their combinations have a greater potential to achieve a more complete description of a forest structure in detail. Notably, despite the envisioned advantages of the synergetic usage of the two types of LiDAR data, there is still considerable uncertainty regarding multiple factors, which mainly include tree detection, trunk diameter and tree height extraction, and the variations in stem density and the terrain of forest areas. How the final estimation results are determined while considering these factors, as well as the sensitivity of these factors to estimations of individual trees’ structures (and volume table construction), should be examined, and the propagation of these errors could be assessed in the future.

According to the BLS data used in this study, only one horizontal-scanning laser scanner was used, so the height of the scanned point cloud could only reach about 9 m, and the point cloud information was difficult to obtain for the parts of trunks below crowns at 9 m or more, so the diameter information of the upper parts of the tree trunks was limited. In future research, a BLS with two laser sensors will be used to obtain more complete point cloud data for the forest in order to obtain a greater tree trunk diameter, which will be more accurate when constructing the taper equations. In addition, the standard tree volume model and the standard volume table obtained with the research method were strong with respect to a specific research area. Our approach was non-destructive, and the growth conditions of the tree species in the state-operated Dongtai Forest Farm were reflected with high precision. However, the applicability and transferability of our approach to individual tree segmentation and the construction of taper models of dawn redwood and poplar tree species in other regions need to be verified. In the future, field data samples will be collected in the whole province, and a standard volume model for planted forests with stronger applicability will be established.

## 5. Conclusions

This study used near-surface LiDAR data (i.e., ULS and BLS) to systematically derive forest structural parameters (tree height, DBH, upper trunk diameter) and to construct standard volume models and corresponding volume tables of two tree species, i.e., dawn redwood and poplar, in a subtropical coastal plantation in Jiangsu Province, China. The fusion of ULS and BLS data provides an advanced technical means and a better approach in order to support forest investigations, since forest structural parameters can be extracted quickly and efficiently without destroying the forest’s trees. In this study, the stem curves of forest stands were obtained directly and quickly, the optimal taper equations were found, and the standard volume equations and stand volumes were further obtained once the structural parameters were accurately derived from LiDAR data. It was found that the individual tree segmentation with ULS and BLS data using a direct point-cloud-oriented clustering segmentation algorithm achieved better segmentation results. At the same time, the extraction of parameters of individual tree structure based on LiDAR data was more accurate. The object-oriented point-cloud-matching algorithm had high accuracy for ULS and BLS point cloud matching. The average error of the location for the dawn redwood plots was 27–36 cm, and the average error of the location for the poplar plots was 54–67 cm. The modified Schumacher model fit the taper equation well. At the same time, the standard volume equation could be used to calculate the standing tree volume more accurately. This study provided new ideas about non-destructive measurement of individual trees’ structural parameters and the construction of volume tables, which could make significant contributions to precision silviculture and sustainable forest management.

## Figures and Tables

**Table 1:** Summary of the structural attributes of the field plots.

Plots	Tree Species	Age	Measured Trees (Trees)	Mean DBH (cm)	Standard Deviation of DBH (cm)	Mean Height (m)	Standard Deviation of Height (m)
a	Dawn redwood	39	12	29.11	5.97	25.30	2.47
b	Dawn redwood	43	26	31.57	3.95	27.30	1.71
c	Dawn redwood	34	20	27.11	6.42	23.22	2.41
d	Poplar	21	9	33.30	10.32	27.84	3.59
e	Poplar	22	12	34.57	5.22	31.46	1.90
f	Poplar	23	11	38.15	7.57	33.16	2.41
g	Poplar	13	16	24.69	5.44	23.20	2.14

**Table 2:** Assessment of the accuracy of the ULS point cloud data segmentation. Nt: the number of treetops detected that existed in the field position; No: the number of trees that were omitted by the algorithm; Nc: the number of detected trees that did not exist in the field; *r*: detection rate; *p*: the precision of detected trees; *F*: overall accuracy.

Plots	Tree Species	Nt	No	Nc	Recall (*r*)	Precision (*p*)	Plots Overall Accuracy (*F*)	Species’ Overall Accuracy (*F*)
a	Dawn redwood	25	2	4	0.93	0.86	0.89	0.90
b	Dawn redwood	45	5	6	0.90	0.89	0.89	
c	Dawn redwood	54	3	5	0.95	0.92	0.93	
d	Poplar	15	3	2	0.83	0.88	0.85	0.88
e	Poplar	19	3	2	0.88	0.90	0.89	
f	Poplar	19	2	3	0.90	0.87	0.88	
g	Poplar	38	3	7	0.92	0.85	0.88	

**Table 3:** Assessment of the accuracy of BLS point cloud data segmentation. Nt: the number of treetops detected that existed in the field position; No: the number of trees that were omitted by the algorithm; Nc: the number of detected trees that did not exist in the field; *r*: detection rate; *p*: the precision of the detected trees; *F*: overall accuracy.

Plots	Tree Species	Nt	No	Nc	Recall (*r*)	Precision (*p*)	Plots Overall Accuracy (*F*)	Spec’es’ Overall Accuracy (*F*)
a	Dawn redwood	26	1	2	0.96	0.92	0.94	0.93
b	Dawn redwood	48	2	6	0.97	0.89	0.92	
c	Dawn redwood	55	2	6	0.96	0.90	0.93	
d	Poplar	17	1	2	0.92	0.89	0.90	0.92
e	Poplar	20	1	1	0.94	0.93	0.93	
f	Poplar	20	1	2	0.96	0.90	0.93	
g	Poplar	39	3	5	0.94	0.89	0.91	

**Table 4:** Assessment of the accuracy of the tree height and DBH extracted from LiDAR data.

	Dawn Redwood				Poplar			
Parameters	R^2^	MAE	RMSE	rRMSE (%)	R^2^	MAE	RMSE	rRMSE (%)
Tree height	0.913	0.605 m	0.849 m	3.00	0.934	0.853 m	1.246 m	4.40
DBH	0.945	1.131 cm	1.300 cm	4.50	0.977	1.162 cm	1.600 cm	4.90

**Table 5:** The distance error of the coarse co-registration of seven plots.

Plots	Tree Species	Stem Density	Distance Error (cm)
a	Dawn redwood	Low	84
b	Dawn redwood	Medium	61
c	Dawn redwood	High	56
d	Poplar	Low	158
r	Poplar	Medium	138
f	Poplar	Medium	159
g	Poplar	High	146

**Table 6:** Assessment of the accuracy of the five models of dawn redwood and poplar.

	Dawn Redwood				Poplar			
Model Id	R^2^	MAE (cm)	RMSE (cm)	rRMSE (%)	R^2^	MAE (cm)	RMSE (cm)	rRMSE (%)
➀	0.906	1.2	1.6	5.9	0.926	1.5	2.3	8.0
➁	0.910	1.1	1.5	5.7	0.936	1.3	2.2	7.5
➂	0.733	2.1	2.8	9.8	0.761	3.4	4.2	13.6
➃	0.907	1.1	1.6	5.8	0.932	1.4	2.2	7.7
➄	0.898	1.2	1.7	6.1	0.919	1.7	2.4	8.5

**Table 7:** Parameters and analysis of the optimal taper function models of dawn redwood and poplar.

	Dawn Redwood				Poplar			
Taper equation coefficients	*a*_0_	*a*_1_	*a*_2_	*a*_3_	*a*_0_	*a*_1_	*a*_2_	*a*_3_
Estimates	0.598	1.900	1.417	1.279	1.501	1.901	1.724	1.854
Standard error	0.144	0.035	0.047	0.082	0.333	0.031	0.063	0.090
95% Confidence lower limit	0.315	1.830	1.325	1.119	0.847	1.839	1.600	1.677
95% Confidence upper limit	0.880	1.969	1.510	1.440	2.155	1.962	1.848	2.032

**Table 8:** Analysis of the accuracy of the standard volume equations of dawn redwood and poplar.

	Dawn Redwood			Poplar		
	R^2^	RMSE (m^3^)	rRMSE (%)	R^2^	RMSE (m^3^)	rRMSE (%)
Tree volume	0.868	0.058	11.2	0.970	0.022	3.3

**Table 9:** Summary of the volumes calculated with Equation (11) and stand volumes calculated with Equation (12) by plot.

Plots	Tree Species	LiDAR-Derived Tree Height (m)	LiDAR-Derived DBH (cm)	LiDAR-Derived Plot Volume (m^3^)	LiDAR-Derived Stand Volume (m^3^/ha)
a	Dawn redwood	25.16	30.39	19.15	212.78
b	Dawn redwood	27.13	31.76	33.42	371.33
c	Dawn redwood	23.17	27.42	16.39	182.11
d	Poplar	27.09	34.15	10.46	116.22
e	Poplar	32.05	34.30	21.75	241.67
f	Poplar	33.44	36.93	24.33	270.33
g	Poplar	23.39	22.93	10.42	115.78

## Supplementary Materials

The following are available online at https://www.mdpi.com/article/10.3390/s21238162/s1, Table S1: Dawn redwood standard volume table compiled with the standard volume equation, Table S2: Poplar standard volume table compiled with the standard volume equation.
