# Supplementary material for: Individual Tree Structural Parameter Extraction and Volume Table Creation Based on Near-Field LiDAR Data: A Case Study in a Subtropical Planted Forest
Source: Sensors (Basel). 2021 Dec 6;21(23):8162. doi: 10.3390/s21238162 (PMC8662440; doi:10.3390/s21238162)
Supplement: Supplementary file 1 [file sensors-21-08162-s001.zip › sensors-1453955-supplementary.pdf]

Supplementary Materials

Table S1. Dawn redwood standard volume table compiled by standard volume equation.

DBH (cm) \ H (m) / V (m³)	1	2	3	4	5	6	7	8	9	10
5	0.00041	0.00091	0.00144	0.00200	0.00258	0.00318	0.00379	0.00441	0.00504	0.00568
6	0.00058	0.00129	0.00204	0.00283	0.00365	0.00449	0.00535	0.00623	0.00713	0.00804
7	0.00078	0.00172	0.00274	0.00380	0.00489	0.00602	0.00718	0.00835	0.00955	0.01077
8	0.00101	0.00222	0.00353	0.00489	0.00631	0.00776	0.00925	0.01077	0.01231	0.01388
9	0.00126	0.00278	0.00441	0.00612	0.00789	0.00971	0.01157	0.01347	0.01540	0.01736
10	0.00154	0.00340	0.00539	0.00748	0.00964	0.01186	0.01413	0.01645	0.01881	0.02121
11	0.00185	0.00407	0.00646	0.00896	0.01155	0.01421	0.01694	0.01972	0.02255	0.02542
12	0.00218	0.00480	0.00762	0.01057	0.01363	0.01677	0.01998	0.02326	0.02660	0.02999
13	0.00254	0.00559	0.00887	0.01231	0.01586	0.01952	0.02327	0.02708	0.03097	0.03491
14	0.00293	0.00644	0.01021	0.01417	0.01826	0.02247	0.02678	0.03118	0.03565	0.04019
15	0.00333	0.00734	0.01164	0.01615	0.02082	0.02562	0.03054	0.03555	0.04065	0.04582
16	0.00377	0.00830	0.01316	0.01826	0.02354	0.02897	0.03452	0.04018	0.04595	0.05180
17	0.00423	0.00931	0.01477	0.02049	0.02641	0.03250	0.03873	0.04509	0.05156	0.05813
18	0.00472	0.01038	0.01646	0.02284	0.02944	0.03623	0.04318	0.05026	0.05747	0.06479
19	0.00523	0.01150	0.01824	0.02531	0.03263	0.04015	0.04785	0.05570	0.06369	0.07180
20	0.00576	0.01268	0.02011	0.02790	0.03597	0.04426	0.05275	0.06140	0.07021	0.07915
21	0.00632	0.01391	0.02206	0.03061	0.03946	0.04856	0.05787	0.06737	0.07703	0.08684
22	0.00690	0.01519	0.02410	0.03344	0.04311	0.05305	0.06322	0.07359	0.08415	0.09487
23	0.00751	0.01653	0.02623	0.03639	0.04691	0.05772	0.06879	0.08008	0.09156	0.10323
24	0.00815	0.01793	0.02844	0.03945	0.05086	0.06258	0.07458	0.08682	0.09928	0.11192
25	0.00880	0.01937	0.03073	0.04263	0.05496	0.06763	0.08060	0.09382	0.10728	0.12095
26	0.00948	0.02087	0.03311	0.04593	0.05921	0.07286	0.08683	0.10108	0.11558	0.13031
27	0.01019	0.02242	0.03557	0.04935	0.06361	0.07828	0.09329	0.10860	0.12417	0.13999
28	0.01092	0.02403	0.03811	0.05288	0.06816	0.08388	0.09996	0.11637	0.13306	0.15001

29	0.01167	0.02568	0.04074	0.05652	0.07286	0.08966	0.10685	0.12439	0.14223	0.16035
30	0.01245	0.02739	0.04345	0.06028	0.07771	0.09563	0.11396	0.13267	0.15170	0.17102
31	0.01325	0.02915	0.04625	0.06416	0.08270	0.10177	0.12129	0.14119	0.16145	0.18201
32	0.01407	0.03097	0.04912	0.06815	0.08785	0.10810	0.12883	0.14997	0.17149	0.19333
33	0.01492	0.03283	0.05208	0.07225	0.09314	0.11461	0.13659	0.15900	0.18181	0.20497

Cont.

DBH (cm) \ V (m^3) \ H (m)	11	12	13	14	15	16	17	18	19	20
5	0.00633	0.00699	0.00766	0.00833	0.00901	0.00970	0.01039	0.01109	0.01180	0.01251
6	0.00896	0.00989	0.01083	0.01178	0.01275	0.01372	0.01470	0.01569	0.01668	0.01768
7	0.01200	0.01325	0.01452	0.01579	0.01708	0.01839	0.01970	0.02102	0.02236	0.02370
8	0.01547	0.01708	0.01871	0.02036	0.02202	0.02370	0.02539	0.02709	0.02881	0.03055
9	0.01935	0.02136	0.02340	0.02546	0.02754	0.02964	0.03176	0.03389	0.03604	0.03821
10	0.02364	0.02610	0.02859	0.03110	0.03364	0.03621	0.03879	0.04140	0.04403	0.04668
11	0.02833	0.03128	0.03426	0.03728	0.04032	0.04340	0.04650	0.04962	0.05277	0.05594
12	0.03342	0.03690	0.04042	0.04398	0.04757	0.05120	0.05485	0.05854	0.06226	0.06600
13	0.03891	0.04297	0.04706	0.05120	0.05539	0.05961	0.06386	0.06816	0.07248	0.07684
14	0.04480	0.04946	0.05418	0.05895	0.06376	0.06862	0.07352	0.07846	0.08344	0.08846
15	0.05107	0.05639	0.06177	0.06720	0.07269	0.07823	0.08382	0.08945	0.09513	0.10085
16	0.05774	0.06375	0.06982	0.07597	0.08217	0.08844	0.09475	0.10112	0.10754	0.11400
17	0.06478	0.07153	0.07835	0.08524	0.09221	0.09923	0.10632	0.11347	0.12067	0.12792
18	0.07222	0.07973	0.08734	0.09502	0.10278	0.11062	0.11852	0.12648	0.13451	0.14260
19	0.08003	0.08836	0.09679	0.10530	0.11390	0.12258	0.13134	0.14017	0.14906	0.15802
20	0.08822	0.09740	0.10669	0.11608	0.12556	0.13513	0.14478	0.15452	0.16432	0.17420
21	0.09679	0.10687	0.11706	0.12736	0.13776	0.14826	0.15885	0.16952	0.18028	0.19112
22	0.10574	0.11674	0.12787	0.13913	0.15049	0.16196	0.17353	0.18519	0.19694	0.20878
23	0.11505	0.12703	0.13914	0.15139	0.16375	0.17623	0.18882	0.20151	0.21430	0.22718
24	0.12474	0.13773	0.15086	0.16414	0.17754	0.19108	0.20472	0.21848	0.23235	0.24631
25	0.13480	0.14884	0.16303	0.17738	0.19186	0.20649	0.22123	0.23610	0.25109	0.26618
26	0.14523	0.16035	0.17564	0.19110	0.20671	0.22246	0.23835	0.25437	0.27051	0.28677
27	0.15603	0.17227	0.18870	0.20530	0.22207	0.23900	0.25607	0.27328	0.29062	0.30809
28	0.16719	0.18460	0.20220	0.21999	0.23796	0.25610	0.27439	0.29283	0.31141	0.33013
29	0.17872	0.19732	0.21614	0.23516	0.25437	0.27375	0.29331	0.31302	0.33288	0.35289
30	0.19061	0.21045	0.23052	0.25081	0.27129	0.29197	0.31282	0.33385	0.35503	0.37637
31	0.20286	0.22398	0.24534	0.26693	0.28873	0.31073	0.33293	0.35531	0.37785	0.40057
32	0.21548	0.23791	0.26060	0.28353	0.30668	0.33006	0.35363	0.37740	0.40135	0.42547
33	0.22845	0.25223	0.27629	0.30060	0.32515	0.34993	0.37492	0.40012	0.42551	0.45109

Cont.

DBH (cm) \ H (m) V (m^3)	21	22	23	24	25	26	27	28	29	30
5	0.01322	0.01394	0.01466	0.01539	0.01612	0.01686	0.01760	0.01834	0.01909	0.01984
6	0.01869	0.01971	0.02073	0.02176	0.02280	0.02384	0.02488	0.02593	0.02699	0.02805
7	0.02505	0.02642	0.02779	0.02917	0.03055	0.03195	0.03335	0.03476	0.03618	0.03760
8	0.03229	0.03405	0.03581	0.03759	0.03938	0.04117	0.04298	0.04480	0.04662	0.04846
9	0.04039	0.04258	0.04479	0.04702	0.04925	0.05150	0.05376	0.05603	0.05832	0.06061
10	0.04934	0.05202	0.05472	0.05744	0.06017	0.06291	0.06568	0.06845	0.07124	0.07404
11	0.05914	0.06235	0.06559	0.06884	0.07211	0.07541	0.07871	0.08204	0.08538	0.08874
12	0.06977	0.07356	0.07738	0.08122	0.08508	0.08896	0.09286	0.09679	0.10073	0.10469
13	0.08123	0.08564	0.09009	0.09456	0.09905	0.10357	0.10812	0.11269	0.11728	0.12189
14	0.09351	0.09859	0.10371	0.10885	0.11403	0.11923	0.12447	0.12972	0.13501	0.14032
15	0.10660	0.11240	0.11823	0.12410	0.13000	0.13593	0.14190	0.14790	0.15392	0.15997
16	0.12051	0.12706	0.13366	0.14029	0.14696	0.15367	0.16041	0.16719	0.17400	0.18084
17	0.13522	0.14258	0.14997	0.15742	0.16490	0.17243	0.17999	0.18760	0.19524	0.20292
18	0.15074	0.15893	0.16718	0.17547	0.18382	0.19221	0.20064	0.20912	0.21764	0.22620
19	0.16704	0.17613	0.18526	0.19446	0.20371	0.21300	0.22235	0.23175	0.24119	0.25067
20	0.18414	0.19415	0.20423	0.21436	0.22456	0.23481	0.24511	0.25547	0.26588	0.27633
21	0.20203	0.21301	0.22407	0.23519	0.24637	0.25761	0.26892	0.28028	0.29170	0.30318
22	0.22070	0.23270	0.24477	0.25692	0.26914	0.28142	0.29377	0.30618	0.31866	0.33119
23	0.24015	0.25321	0.26634	0.27956	0.29286	0.30622	0.31966	0.33317	0.34674	0.36038
24	0.26038	0.27453	0.28878	0.30311	0.31752	0.33201	0.34658	0.36123	0.37595	0.39073
25	0.28138	0.29667	0.31207	0.32755	0.34313	0.35879	0.37453	0.39036	0.40626	0.42224
26	0.30314	0.31963	0.33621	0.35289	0.36967	0.38655	0.40351	0.42056	0.43770	0.45491
27	0.32568	0.34339	0.36120	0.37913	0.39716	0.41528	0.43351	0.45183	0.47023	0.48873
28	0.34898	0.36795	0.38704	0.40625	0.42557	0.44499	0.46452	0.48415	0.50388	0.52370
29	0.37304	0.39332	0.41373	0.43426	0.45491	0.47567	0.49655	0.51753	0.53862	0.55980
30	0.39786	0.41949	0.44126	0.46315	0.48518	0.50732	0.52959	0.55196	0.57445	0.59705
31	0.42344	0.44646	0.46962	0.49293	0.51637	0.53993	0.56363	0.58745	0.61138	0.63543
32	0.44976	0.47422	0.49882	0.52358	0.54847	0.57351	0.59868	0.62397	0.64939	0.67494
33	0.47684	0.50277	0.52886	0.55510	0.58150	0.60804	0.63472	0.66154	0.68849	0.71557

Table S2. Poplar standard volume table compiled by standard volume equation.

DBH (cm) \ H (m) V (m^3)	1	2	3	4	5	6	7	8	9	10
5	0.00092	0.00169	0.00240	0.00308	0.00374	0.00439	0.00501	0.00563	0.00624	0.00684
6	0.00130	0.00238	0.00339	0.00436	0.00529	0.00620	0.00709	0.00797	0.00883	0.00967
7	0.00175	0.00320	0.00455	0.00584	0.00709	0.00831	0.00951	0.01068	0.01183	0.01297
8	0.00225	0.00412	0.00586	0.00753	0.00914	0.01072	0.01225	0.01376	0.01525	0.01671

9	0.00282	0.00515	0.00733	0.00942	0.01144	0.01341	0.01533	0.01722	0.01908	0.02091
10	0.00345	0.00630	0.00896	0.01151	0.01398	0.01638	0.01873	0.02104	0.02331	0.02554
11	0.00413	0.00755	0.01074	0.01380	0.01675	0.01963	0.02245	0.02521	0.02793	0.03062
12	0.00487	0.00891	0.01267	0.01628	0.01976	0.02316	0.02649	0.02975	0.03296	0.03612
13	0.00567	0.01037	0.01476	0.01895	0.02301	0.02697	0.03084	0.03464	0.03838	0.04206
14	0.00653	0.01194	0.01699	0.02182	0.02649	0.03105	0.03550	0.03988	0.04418	0.04842
15	0.00745	0.01361	0.01937	0.02488	0.03021	0.03540	0.04048	0.04547	0.05037	0.05521
16	0.00842	0.01539	0.02190	0.02812	0.03415	0.04002	0.04576	0.05140	0.05695	0.06242
17	0.00945	0.01727	0.02457	0.03156	0.03832	0.04491	0.05135	0.05768	0.06391	0.07004
18	0.01053	0.01925	0.02739	0.03518	0.04272	0.05006	0.05725	0.06430	0.07124	0.07808
19	0.01167	0.02133	0.03036	0.03899	0.04735	0.05548	0.06345	0.07126	0.07895	0.08653
20	0.01287	0.02352	0.03347	0.04298	0.05219	0.06117	0.06995	0.07856	0.08704	0.09539
21	0.01412	0.02580	0.03672	0.04716	0.05727	0.06711	0.07674	0.08620	0.09550	0.10467
22	0.01542	0.02819	0.04011	0.05152	0.06256	0.07332	0.08384	0.09417	0.10433	0.11434
23	0.01678	0.03068	0.04365	0.05607	0.06808	0.07978	0.09123	0.10247	0.11353	0.12443
24	0.01820	0.03326	0.04733	0.06079	0.07382	0.08650	0.09892	0.11110	0.12309	0.13491
25	0.01967	0.03595	0.05115	0.06570	0.07977	0.09348	0.10690	0.12007	0.13303	0.14580
26	0.02119	0.03873	0.05511	0.07078	0.08595	0.10072	0.11518	0.12936	0.14332	0.15708
27	0.02277	0.04161	0.05921	0.07605	0.09234	0.10821	0.12374	0.13899	0.15398	0.16877
28	0.02440	0.04459	0.06345	0.08149	0.09895	0.11596	0.13260	0.14894	0.16501	0.18085
29	0.02608	0.04766	0.06782	0.08711	0.10578	0.12396	0.14175	0.15921	0.17639	0.19332
30	0.02781	0.05084	0.07234	0.09291	0.11282	0.13221	0.15118	0.16981	0.18813	0.20619
31	0.02960	0.05411	0.07699	0.09889	0.12007	0.14071	0.16091	0.18073	0.20023	0.21945
32	0.03145	0.05747	0.08178	0.10504	0.12754	0.14947	0.17092	0.19197	0.21269	0.23311
33	0.03334	0.06093	0.08671	0.11137	0.13523	0.15847	0.18121	0.20354	0.22550	0.24715

Cont.

H (m) / V (m^3) / DBH (cm)	11	12	13	14	15	16	17	18	19	20
5	0.00743	0.00801	0.00859	0.00917	0.00973	0.01029	0.01085	0.01140	0.01195	0.01250
6	0.01051	0.01133	0.01215	0.01296	0.01376	0.01456	0.01535	0.01613	0.01691	0.01768
7	0.01409	0.01519	0.01629	0.01738	0.01845	0.01952	0.02057	0.02162	0.02266	0.02370
8	0.01816	0.01959	0.02100	0.02240	0.02378	0.02515	0.02652	0.02787	0.02921	0.03054
9	0.02271	0.02450	0.02627	0.02802	0.02975	0.03147	0.03317	0.03486	0.03654	0.03821
10	0.02775	0.02993	0.03209	0.03423	0.03635	0.03845	0.04053	0.04259	0.04465	0.04668
11	0.03326	0.03588	0.03847	0.04103	0.04357	0.04608	0.04858	0.05105	0.05351	0.05596
12	0.03925	0.04233	0.04539	0.04841	0.05140	0.05437	0.05732	0.06024	0.06314	0.06602
13	0.04570	0.04929	0.05284	0.05636	0.05985	0.06331	0.06674	0.07014	0.07352	0.07687
14	0.05261	0.05675	0.06084	0.06489	0.06891	0.07288	0.07683	0.08075	0.08464	0.08850
15	0.05998	0.06470	0.06937	0.07399	0.07856	0.08310	0.08760	0.09207	0.09650	0.10090

16	0.06781	0.07314	0.07842	0.08364	0.08882	0.09395	0.09903	0.10408	0.10910	0.11408
17	0.07610	0.08208	0.08800	0.09386	0.09967	0.10542	0.11113	0.11680	0.12242	0.12801
18	0.08483	0.09150	0.09810	0.10463	0.11111	0.11752	0.12389	0.13020	0.13647	0.14270
19	0.09401	0.10141	0.10872	0.11596	0.12313	0.13024	0.13730	0.14430	0.15125	0.15815
20	0.10364	0.11179	0.11985	0.12784	0.13574	0.14358	0.15136	0.15908	0.16674	0.17435
21	0.11371	0.12266	0.13150	0.14026	0.14894	0.15754	0.16607	0.17454	0.18294	0.19129
22	0.12423	0.13400	0.14366	0.15323	0.16271	0.17210	0.18143	0.19068	0.19986	0.20898
23	0.13518	0.14581	0.15633	0.16674	0.17705	0.18728	0.19742	0.20749	0.21748	0.22741
24	0.14657	0.15810	0.16950	0.18079	0.19197	0.20306	0.21406	0.22497	0.23581	0.24657
25	0.15840	0.17086	0.18318	0.19538	0.20747	0.21945	0.23133	0.24313	0.25484	0.26647
26	0.17066	0.18408	0.19736	0.21050	0.22353	0.23644	0.24924	0.26195	0.27456	0.28709
27	0.18336	0.19778	0.21204	0.22616	0.24015	0.25402	0.26778	0.28143	0.29499	0.30845
28	0.19648	0.21193	0.22722	0.24235	0.25734	0.27220	0.28695	0.30158	0.31610	0.33053
29	0.21004	0.22655	0.24289	0.25907	0.27509	0.29098	0.30674	0.32238	0.33791	0.35333
30	0.22402	0.24163	0.25906	0.27631	0.29341	0.31035	0.32716	0.34384	0.36040	0.37685
31	0.23843	0.25718	0.27572	0.29409	0.31228	0.33031	0.34820	0.36596	0.38358	0.40109
32	0.25326	0.27318	0.29288	0.31238	0.33171	0.35086	0.36987	0.38872	0.40745	0.42604
33	0.26852	0.28963	0.31052	0.33120	0.35169	0.37200	0.39215	0.41214	0.43199	0.45170

Cont.

H (m) / V (m^3) / DBH (cm)	11	22	23	24	25	26	27	28	29	30
5	0.01304	0.01358	0.01412	0.01465	0.01518	0.01570	0.01623	0.01675	0.01727	0.01779
6	0.01844	0.01921	0.01996	0.02072	0.02147	0.02221	0.02295	0.02369	0.02442	0.02516
7	0.02472	0.02575	0.02676	0.02777	0.02877	0.02977	0.03077	0.03176	0.03274	0.03372
8	0.03187	0.03319	0.03449	0.03579	0.03709	0.03838	0.03966	0.04093	0.04220	0.04346
9	0.03987	0.04151	0.04315	0.04478	0.04640	0.04801	0.04961	0.05120	0.05279	0.05437
10	0.04871	0.05072	0.05272	0.05471	0.05669	0.05865	0.06061	0.06256	0.06450	0.06643
11	0.05838	0.06079	0.06319	0.06557	0.06795	0.07030	0.07265	0.07499	0.07731	0.07962
12	0.06888	0.07173	0.07456	0.07737	0.08017	0.08295	0.08572	0.08847	0.09122	0.09395
13	0.08020	0.08352	0.08681	0.09009	0.09334	0.09658	0.09981	0.10301	0.10621	0.10939
14	0.09234	0.09615	0.09994	0.10371	0.10746	0.11119	0.11491	0.11860	0.12228	0.12594
15	0.10528	0.10963	0.11395	0.11825	0.12252	0.12678	0.13101	0.13522	0.13941	0.14358
16	0.11902	0.12394	0.12882	0.13368	0.13852	0.14333	0.14811	0.15287	0.15761	0.16233
17	0.13356	0.13908	0.14456	0.15001	0.15544	0.16083	0.16620	0.17154	0.17686	0.18216

18	0.14889	0.15504	0.16115	0.16723	0.17328	0.17929	0.18528	0.19123	0.19716	0.20306
19	0.16501	0.17182	0.17860	0.18534	0.19204	0.19870	0.20533	0.21193	0.21850	0.22505
20	0.18191	0.18942	0.19689	0.20432	0.21170	0.21905	0.22636	0.23364	0.24088	0.24809
21	0.19959	0.20783	0.21603	0.22417	0.23228	0.24034	0.24836	0.25635	0.26429	0.27221
22	0.21804	0.22705	0.23600	0.24490	0.25376	0.26256	0.27133	0.28005	0.28873	0.29738
23	0.23727	0.24707	0.25681	0.26650	0.27613	0.28572	0.29525	0.30474	0.31419	0.32360
24	0.25726	0.26789	0.27845	0.28895	0.29940	0.30979	0.32013	0.33042	0.34067	0.35087
25	0.27802	0.28950	0.30092	0.31227	0.32356	0.33479	0.34597	0.35709	0.36816	0.37918
26	0.29954	0.31191	0.32421	0.33644	0.34861	0.36071	0.37275	0.38473	0.39666	0.40853
27	0.32182	0.33511	0.34833	0.36147	0.37454	0.38754	0.40047	0.41335	0.42616	0.43892
28	0.34486	0.35910	0.37326	0.38734	0.40135	0.41528	0.42914	0.44293	0.45666	0.47033
29	0.36865	0.38387	0.39901	0.41406	0.42903	0.44392	0.45874	0.47349	0.48817	0.50278
30	0.39319	0.40943	0.42557	0.44163	0.45759	0.47348	0.48928	0.50501	0.52066	0.53625
31	0.41848	0.43576	0.45294	0.47003	0.48702	0.50393	0.52075	0.53749	0.55415	0.57074
32	0.44451	0.46287	0.48112	0.49927	0.51732	0.53528	0.55315	0.57093	0.58863	0.60625
33	0.47129	0.49076	0.51011	0.52935	0.54849	0.56753	0.58647	0.60532	0.62409	0.64277
